# Supplementary material for: Identification and functional analysis of non-coding regulatory small RNA FenSr3 in Bacillus amyloliquefaciens LPB-18
Source: PeerJ. 2023 May 15;11:e15236. doi: 10.7717/peerj.15236 (PMC10194069; doi:10.7717/peerj.15236)
Supplement: Supplemental Information 4 [file peerj-11-15236-s004.zip › KO/CK-vs-T1_map/map04625.html]

KEGG PATHWAY: C-type lectin receptor signaling pathway - Reference pathway


|  |  |
| --- | --- |
| **C-type lectin receptor signaling pathway - Reference pathway** |  |

[
Pathway menu
| Organism menu
| Pathway entry
| Show description
| User data mapping
]

C-type lectin receptors (CLRs) are a large superfamily of proteins characterized by the presence of one or more C-type lectin-like domains (CTLDs). CLRs function as pattern-recognition receptors (PRRs) for pathogen-derived ligands in dendric cells, macrophages, neutrophils, etc., such as Dectin-1 and Dectin-2 for recognition of fungi-derived B-glucan and high mannose-type carbohydrates. Upon ligand binding, CLRs stimulate intracellular signaling cascades that induce the production of inflammatory cytokines and chemokines, consequently triggering innate and adaptive immunity to pathogens.

|  |  |  |
| --- | --- | --- |
| Reference pathway | 184% 150% 122% 100% 82% 67% 55% | 图片下载 |
